# Time‐Resolved Fluorescence and Diffuse Reflectance (TRF‐DR) Spectroscopy for Heterogeneous Breast Tissue Classification and Tumor Margin Assessment

**DOI:** 10.1002/jbio.70230

**Published:** 2026-02-01

**Authors:** Jigar Lad, Erica Dao, Gabriella Gohla, Peter Lovrics, Qiyin Fang, Thomas J. Farrell, Michael J. Farquharson

**Affiliations:** ^1^ Department of Physics and Astronomy McMaster University Hamilton Ontario Canada; ^2^ St. Joseph's Hospital Hamilton Ontario Canada; ^3^ Department of Pathology and Molecular Medicine McMaster University Hamilton Ontario Canada; ^4^ Department of Surgery McMaster University Hamilton Ontario Canada; ^5^ Department of Engineering Physics McMaster University Hamilton Ontario Canada; ^6^ Faculty of Health Science McMaster University Hamilton Ontario Canada; ^7^ School of Interdisciplinary Science McMaster University Hamilton Ontario Canada

**Keywords:** breast cancer, breast conserving surgery, diffuse reflectance, fluorescence, machine learning classification, optical spectroscopy, tissue heterogeneity

## Abstract

Re‐excision rates remain high for early‐stage breast cancer patients due to challenges in margin delineation during surgery, such as poorly defined tumor boundaries. Our group has developed a time‐resolved fluorescence and diffuse reflectance (TRF‐DR) spectroscopy system to assess tumor boundaries where tissue composition is heterogeneous. This study assesses system feasibility for classifying tumor in heterogeneous regions using weighted logistic regression and principal component analysis (PCA). A total of 4818 measurements from 73 frozen patient ex vivo samples were evaluated. Tissue measurement areas (1 mm × 1 mm) were pathologist‐assigned percentages of low (< 25%), medium (25%–< 75%), and high (≥ 75%) for tumor, fibroglandular, or adipose composition. The model trained with four principal components (PCs) achieved sensitivity of 77% and specificity of 68%, comparable to current clinical techniques while being fast, portable, and economical. The TRF‐DR system with weighted logistic regression demonstrates potential as an intraoperative margin assessment tool.

## Graphical abstract

This study demonstrates our time‐resolved fluorescence and diffuse reflectance spectroscopy system can classify breast tumor in heterogeneous tissue. It achieved sensitivity and specificity comparable to other techniques and highlights the effect of heterogeneous measurements on performance. These results support the potential of this system as an intraoperative margin assessment tool.

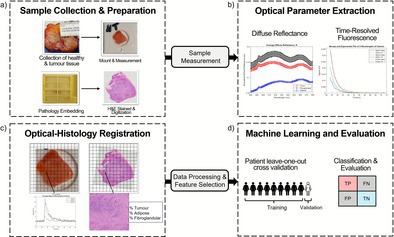

## 1 Introduction

Breast conserving surgery (i.e., lumpectomy) is the recommended approach for early‐stage (i.e., Stage I or II) breast cancer patients [1, 2]. Approximately 50%–60% of newly diagnosed patients undergo lumpectomies [2, 3] with the remaining opting for mastectomies [3]. However, re‐excision rates vary as much as 15%–60% [2, 4, 5, 6], due to inconsistent negative margin width definitions, cancer subtype, and institutional differences [1, 2, 7, 8]. Current protocol involves confirming margin status post‐operatively by a pathologist which can take days to reach a diagnosis [4, 6]. The delay in conveying margin status presents a significant detriment to patient well‐being and hospital resources while potentially delaying adjuvant radiotherapy [5, 9, 10]. It is known that positive (tumor on ink) and close margins (< 1–2 mm) are strong indicators for local recurrence [1, 2, 5, 6, 11]. Therefore, more quantitative intraoperative methods are needed to help identify positive margins and ultimately reduce the need for reoperation.

A variety of methods to delineate tumor margin have been developed and implemented clinically to varying levels of success. Intraoperative pathology approaches include techniques such as frozen sectioning and imprint cytology. These methods typically offer high sensitivities and specificities but can be prone to artifacts, and are timely and expensive to implement [4, 6]. Techniques such as specimen radiography and intraoperative ultrasound are used quite often for inserting surgical clips or wires as fiducial markers for margin interpretation [4, 6]. The former remains popular due to its ease of workflow implementation and image interpretation; however, its low sensitivity has made it difficult to identify positive margins in denser breast tissue [4, 6]. Ultrasound is better for denser breasts while also being far more cost‐effective; however, it may be less suitable for smaller margin widths [4].

Emerging technologies are currently being developed with some reaching commercial deployment but are still not yet widespread. Some of these include but are not limited to: bioimpedance (ClearEdge) and radiofrequency spectroscopy (MarginProbe [5]); mass spectrometry (MasSpec Pen, iKnife [10]); optical coherence tomography (OTIS, SELENE [10]); whole slide imaging (Histolog scanner [12]); Raman spectroscopy [4, 13]; fluorescence‐guided surgery using molecular dyes (LumiSystem [10], indocyanine green [14], 5‐ALA [15]); and photoacoustic imaging [4, 13]. These modalities offer improved measurement acquisition times, ranging from seconds to minutes depending on whether they are probe‐based or imaging‐based, with varying levels of clinical workflow compatibility. However, general limitations and trade‐offs include tissue penetration depth, field‐of‐view, signal‐to‐noise ratio (SNR), and spatial resolution [13, 16]. For example, probe‐based techniques may offer faster single‐point acquisitions and can allow for intracavity measurements; however, they may be prone to user error when compared with imaging‐based methods [16]. Specific cases such as iKnife, although offering high performance, are destructive technique [17]. ClearEdge requires a baseline measurement of the patients' normal breast tissue in order for its algorithm to work, which introduces potential error [17]. The use of exogenous fluorescence agents is invasive and many approved dyes do not have specific breast tumortumor uptake [4, 13]. MarginProbe, although clinically implemented, its impact on re‐excision rates has been disputed and varies institutionally [18].

Our group has developed a dual‐optical spectroscopy system which combines time‐resolved fluorescence (TRF) and diffuse reflectance (DR), with the former focusing on endogenous fluorophores (i.e., reduced nicotinamide adenine dinucleotide [NADH], oxidized flavin adenine dinucleotide [FAD], and collagen) [19, 20, 21, 22]. Prior work using the TRF‐DR system with a decision tree classifier achieved tumor sensitivity of 85.6% and specificity of 95.3% [19, 20]. This is comparable to other similarly designed DR [16, 23, 24, 25, 26, 27, 28] and fluorescence‐based [29, 30, 31, 32, 33] systems, and other clinical, imaging, and pathology techniques [4, 5, 6, 10]. However, a key limitation of these prior studies was that the final conclusions were based on regions‐of‐interest (ROIs) with homogeneous composition (i.e., 100% tumor, fibroglandular, adipose). This extends to literature as reported results often only assess homogeneous regions, with limited studies investigating the tissue‐area percentages associated with measurements [24, 28, 32, 34]. Studies that performed multi‐tissue classification stratify different malignant lesions (e.g., invasive ductal carcinoma [IDC], invasive lobular carcinoma, ductal‐carcinoma in situ [DCIS]) and benign lesions (fibrocystic change, fibroadenoma) from normal tissue (glandular, adipose) [24, 25, 26, 27, 28, 29, 30, 32]; however, samples with heterogeneous compositions were often discarded from analyses or denoted a specific class if it surpassed an arbitrary threshold [25, 27, 28, 30, 32, 35]. This presents an ideal situation that may not be representative of the glandular‐to‐tumor tissue transition observed at the margin in clinical practice [28]. By omitting heterogeneous regions from analyses, these preclinical studies fail to more accurately represent the system's tissue characterization capabilities as it introduces bias among other confounding factors (e.g., ambient light, increased presence of blood, variations in pressure during tissue‐probe contact), making it difficult to assess system feasibility for intraoperative implementation [34]. It has also been cited as a limitation for other clinical techniques in development [10]. Therefore, assessing heterogeneous ROIs is critical for the development of optical techniques for tumor margin delineation.

The current study assesses the effects of heterogeneous compositions on optical features using machine learning by building on the simpler homogeneous analysis [19, 20] using a weighted logistic regressor with pathologist assigned tissue labels and tissue‐area percentages. With these results, we aim to bring our system closer toward a clinical intraoperative tool capable of assisting surgeons and pathologists in decision making.

## 2 Materials and Methods

### 2.1 Patient Sample Handling and Histology

**FIGURE 1:**
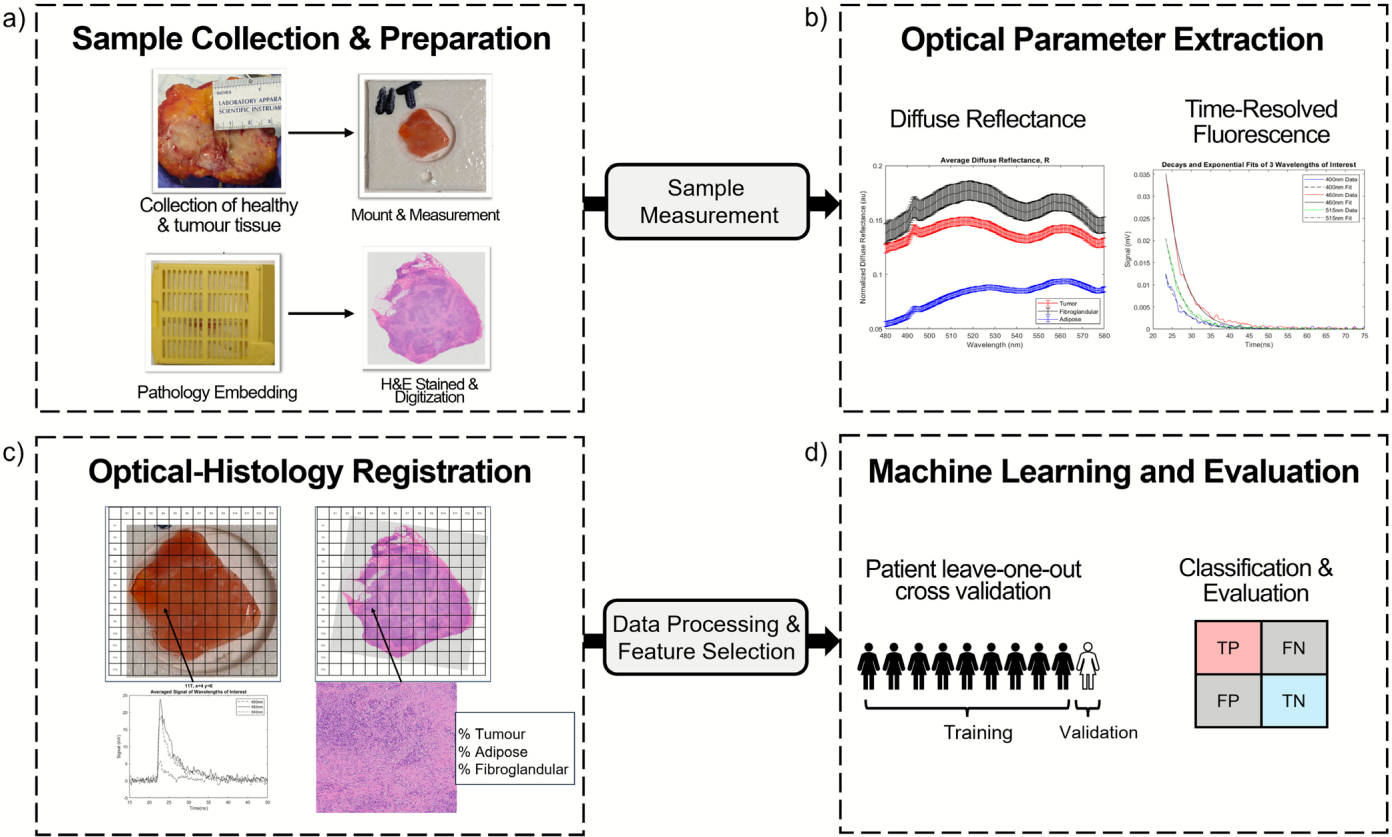
Overview of experimental workflow. (a) Sample preparation and collection. Breast specimens were collected by the breast surgeon. Tumor and healthy tissues were dissected by pathology technicians. After the measurements, samples were embedded, processed, stained, and digitized. (b) Optical parameter extraction. Performed offline to extract corresponding TRF‐DR features. (c) Optical‐histology registration. The white‐light sample image and the digitized hematoxylin and eosin (H&E) image underwent manual rigid registration to correlate TRF‐DR features with pathologist annotations. (d) Machine learning and evaluation. The TRF‐DR features are processed and selected for model training (patient leave‐one‐out cross validation) and evaluated (confusion matrix).

Study approval for sample collection and analysis was granted by the Hamilton Integrated Research Ethics Board at St. Joseph's Hospital, Hamilton, Ontario. This study used an archival dataset of 80 patients with tumor and normal matched‐pair ex vivo samples collected from 2017 to 2020. All frozen samples were collected, processed and analyzed from 2020 to 2022. The specimen was excised from patients undergoing either lumpectomy or mastectomy. Patients treated with (neo)adjuvant radiotherapy were excluded. Tissue samples were extracted from the excised lump post‐surgery and prior to additional inking. Tumor samples were taken from near the tumor core while normal tissue samples were excised from the margin (Figure 1a). A pathology report was provided for each patient consisting of the following information: age, menopausal status, tumor type, invasive tumor grade, presence of carcinoma in situ, primary tumor stage, nodal status, receptor status (see Data S1).

Samples were collected and kept frozen at −80°C on McMaster University campus. For measurement, samples were thawed, cut to fit into a 12.7 mm (diameter) × 1 mm (depth) sample holder made of polylactic acid and wrapped in Ultralene film. Once measured, samples were wrapped tightly in lens paper and processed using standard histopathology protocol (Figure 1a). The corresponding digitized H&E slide was manually registered with a white‐light phone camera photo of the sample using a 13 mm × 13 mm grid overlay (Figure 1c). The pathologist, blinded to the white‐light photo and optical data, used the H&E image to classify each region as either fibroglandular, adipose, or tumor with an associated tissue‐area percentage (Figure 1c). Additional details on sample preparation and histology can be found in previous studies [19, 20].

### 2.2 Measurement System and Acquisition

**FIGURE 2:**
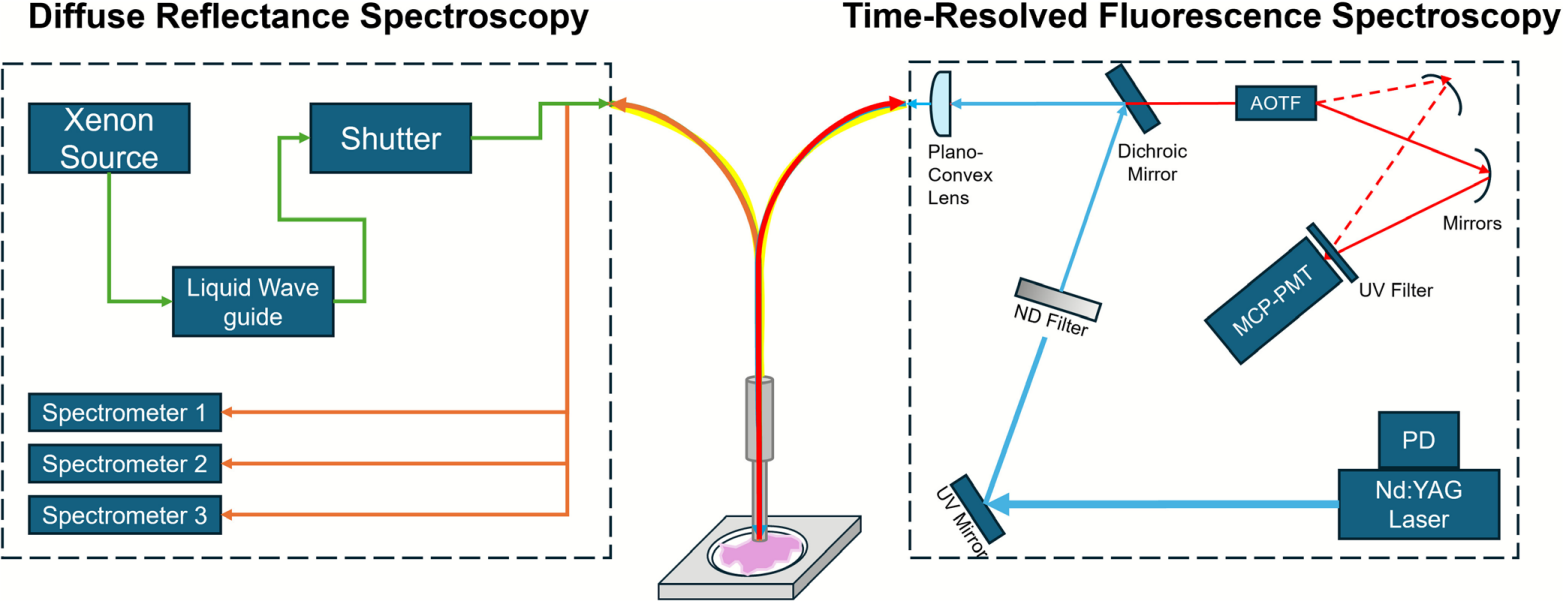
Schematic illustrating the TRF and DR spectroscopy subsystems. AOTF, acousto‐optical tunable filter; MCP‐PMT, microchannel plate photomultiplier tube; ND, neutral density; PD, photodiode. DR subsystem: Upon point‐acquisition, the shutter opens allowing for the broadband light (green arrow) to couple into the DR source fiber where the collected reflectance signal (orange arrow) is detected by the spectrometers corresponding to different sampling distances. TRF subsystem: After DR measurement, the Nd:YAG laser turns on (blue arrow) where the beam is reflected off a UV mirror, passes through a graded ND filter and further reflected by a dichroic mirror for beam attenuation (thinner blue arrow). The light is then focused and coupled by the plano‐convex lens into the probe. The fluorescence signal (red arrow) returns and enters the AOTF for wavelength selection. The ordinary (solid red arrow) and extraordinary (dashed red arrow) waves reflect off concave mirrors and focused onto the MCP‐PMT. The signal is temporarily stored by a digitizer before being sent to the system computer for offline processing.

Measurements were performed using the previously reported TRF‐DR system [19, 21]. A schematic is shown in Figure 2. The TRF subsystem has a single central source and collection fiber (Optran WF, Ceramoptec, NA = 0.12, 400 μm). A 355 nm Nd:YAG pulsed laser source is used for excitation (PNV‐M02510‐130, Teem Photonics, France, 1 kHz, 300 ps FWHM, 30 μJ). Fluorescence signal is passed through the AOTF (TEAF5‐0.36‐0.52‐S‐MSD, Brimrose) for wavelength selection (380–570 nm, 5 nm increments), measured by the MCP‐PMT (R5916‐50, Hamamatsu Photonics, −2000 V), before being amplified (C5594‐12, Hamamatsu Photonics, 50 kHz to 1.5 GHz, 36 dB) and digitized (ADQ7DC, 14 bit, 10 GS/s, 2.5 GHz bandwidth, SP Devices, Linköping, Sweden). To increase SNR, the laser sends 11 pulses per wavelength across the full emission range. The DR subsystem has a single source fiber positioned near the probe perimeter and 11 collection fibers arranged in three rows placed at 0.32 mm, 0.64 mm, and 1.5 mm radial distance from the source fiber. A continuous wave broadband Xenon lamp is used (ASB‐XE‐175BF, spectral products, Putnam, Connecticut, United States, 150–200 W, 320–700 nm) and signal is collected with three diffraction‐grating based spectrometers (BW‐UVIS 600 g/mm, 50 μm slit, StellarNet) corresponding to each collection row. Both modalities are combined into a single 3 mm diameter custom bifurcated fiber bundle made of epoxy resin. System timing is controlled via the pulse delay generator (QC9522, Quantum Composers) and is operated with custom Visual Studio 2015 software. Samples were raster scanned using a motorized translational stage (XLS‐1‐80‐1250, Xeryon, Leuven, Belgium) with the probe ~1 mm from the tissue surface. Stage translations were performed in 1 mm × 1 mm increments to form a 13 mm × 13 mm sample measurement area (Figure 1c). Each acquisition took ~5 s (accounting for data storage and stage translation) for a total sample acquisition time of 15 mins. Additional details on system components can be found in previous studies [19, 21, 36, 37].

### 2.3 Optical Parameter Extraction

Optical parameter extraction was performed using custom‐built code in MATLAB (v2024b, MathWorks, Natick, Massachusetts, United States). For TRF the primary features of interest are the average fluorescence lifetime (*τ*) and normalized fluorescence intensity for the respective fluorophores. This was done by averaging the 11 decays per wavelength resulting in 39 average fluorescence decays per measurement. For each fluorophore, *τ* was calculated by fitting each decay to a single exponential function (Beer–Lambert approximation), solving for *τ* and averaging across the relevant fluorescence emission spectrum (i.e., *τ*_collagen_: 380–440 nm; *τ*_NADH_: 450–500 nm; *τ*_FAD_: 520–570 nm) [19]. The normalized fluorescence intensity was determined by integrating the averaged exponential decay for the relevant fluorophore emission wavelengths and normalizing by the peak NADH signal intensity (i.e., 460 nm) to account for variations in probe‐to‐target distance [19]. DR signal from the second collection fiber row was corrected by subtracting the background measurement (i.e., deionized water) and normalizing by the difference between a 99% reflectance standard (Labsphere Inc.) and background. The corrected reflectance spectra were then compared with an experimental look‐up table consisting of reflectance measurements acquired using phantoms (i.e., India ink and polystyrene microspheres) and associated absorption, $\mu_{a}$, and reduced scattering, $\mu_{s}^{\prime }$ coefficients. The corresponding $\mu_{a}$ and reduced scattering $\mu_{s}^{\prime }$ coeffcients for the measured spectra were determined by reducing the *χ* ^2^ value between the measured sample reflectance intensity and phantom reflectance intensity across all wavelengths (400–700 nm) [19]. Features of interest include DR reflectance intensity at wavelengths 520 and 560 nm, $\mu_{a}$, and $\mu_{s}^{\prime }$ at 540, 560, and 576 nm [19]. Additional details on optical parameter extraction can be found in previous studies [19, 20].

### 2.4 ROI Eligibility Criteria and Inferential Statistics

A total of 15 123 point measurements were acquired from 80 patient samples where regions with pathology annotations from a single observer, tissue‐area percentages summed to 100%, and patient sample pairs from both tumor and no tumor were included for analysis. The first criterion was due to only a few regions with multiple pathologist annotations. In addition, statistical outlier detection was determined with interquartile range (IQR) analysis, and datapoints beyond ±1.5 IQR (i.e., beyond 25% or 75%) were removed. Following IQR analysis, the usable dataset was reduced to 4818 point‐measurements across 73 patients assessed in this study.

### 2.5 Data Preprocessing and Feature Extraction

The optical parameters were transformed using a base‐10 logarithm followed by standardization. This was done to reduce skewness and ensure all features were scaled accordingly. Principal component analysis (PCA) was applied to reduce feature space and the optimal number of principal components (PCs) determined retrospectively, where the fewest PCs required to obtain the best performance were selected. Additional results of model classification performance as a function of the number of PCs can be found in Data S1.

### 2.6 Machine Learning and Performance Evaluation

A weighted logistic regressor was used due to advantages such as suitability for binary classification tasks and smaller datasets, less computationally expensive, and the ability to assess posterior probabilities for predictions. A class weighting scheme was necessary given the imbalanced nature of the dataset (tumor: 1812 ROIs; no tumor: 3006 ROIs). This was assigned automatically using Equation (1):

(1) $$w_{\text{class}}=0.5\times \left(\frac{n_{\text{samples}}}{n_{\text{class}}}\;\right)$$

where $n_{\text{samples}}$ is the total number of samples, $n_{\text{class}}$ is the total number of samples for a class, and $w_{\text{class}}$ is the weight assigned to that class [38] resulting in weights 1.33 (tumor) and 0.80 (no tumor). This is a simple method for dealing with inherent class imbalances while minimizing direct data handling interventions (e.g., random removal of datapoints to force balanced classes). The final probabilities determined for each ROI were then overlayed with pathologist‐assigned tissue‐area percentages to assess the effect of heterogeneous compositions on model predictions. A standard 50% tumor probability decision boundary was used where above this threshold the model would predict tumor. Explanations for how these posterior probabilities are calculated can be found in literature [39]. Model predictions were compared with pathologist annotations via 2 × 2 confusion matrix using Orange Software v3.38.1 (Figure 1d). Training and evaluation were performed on the total dataset and on four subsets: (1) heterogeneous‐only (excluding homogeneous regions); (2) low tumor; (3) medium tumor; and (4) high tumor. The sensitivity and specificity were determined using a patient leave‐one‐out cross validation scheme.

## 3 Results and Discussion

This study analyzed 4818 ROIs from 73 patients. Patient characteristics are summarized in Data S1. Table 1 provides a summary f the overall tumor class makeup, proportion of homogeneous and heterogeneous regions, tissue‐area percentages (stratified into high: ≥ 75%, medium: 25%–< 75%, and low: < 25%), and proportion of tumor and no tumor regions consisting of some fibroglandular and adipose tissue. Of note are the number of tumor ROIs where a class imbalance of 38% tumor to 62% no tumor was observed. Compared with other studies which can vary as much as 16%–71% for tumor to 29%–84% for normal tissue measurements [24, 26, 27, 28, 29, 31], this class imbalance is not as extreme. Although such tissue imbalance is inherent, having insufficient examples of either class (e.g., 80% no tumor) risks biasing the model, in addition to only providing samples of a single tissue type. Therefore, accounting for such imbalance is a crucial step, either in the form of weights or by using models less sensitive to such imbalances (e.g., RUSBoost) [24, 34].

**TABLE 1:** Summary of the number of patients, tissue classification, number of homogeneous and heterogeneous ROIs, and tissue‐area compositions.

Patients	73
Number of ROIs	4818
Tissue classification	
Tumor identified ROIs (% of dataset)	1812 (38)
No tumor identified ROIs (% of dataset)	3006 (62)
Number of homogeneous ROIs (% of dataset)	1583 (33)
Tumor (% of dataset)	652 (14)
Fibroglandular (% of dataset)	93 (2)
Adipose (% of dataset)	838 (17)
Number of heterogeneous ROIs (% of dataset)	3235 (67)
Tumor (% of dataset)	1160 (24)
No tumor (% of dataset)	2075 (43)
Tissue‐area percentage	
Tumor area percentage (% of dataset)	
High (≥ 75%)	893 (19)
Medium (25%–75%)	329 (7)
Low (≤ 25%)	590 (12)
Fibroglandular area percentage (% of dataset)	
High (≥ 75%)	1030 (21)
Medium (25%–75%)	922 (19)
Low (≤ 25%)	1098 (23)
Adipose area percentage (% of dataset)	
High (≥ 75%)	1979 (41)
Medium–high (25%–75%)	822 (17)
Low (≤ 25%)	706 (15)
Percentage of tumor Identified ROIs with any fibroglandular	48
Percentage of tumor Identified ROIs with any adipose	30
Percentage of no tumor Identified ROIs with any fibroglandular	72
Percentage of no tumor Identified ROIs with any adipose	97

**FIGURE 3:**
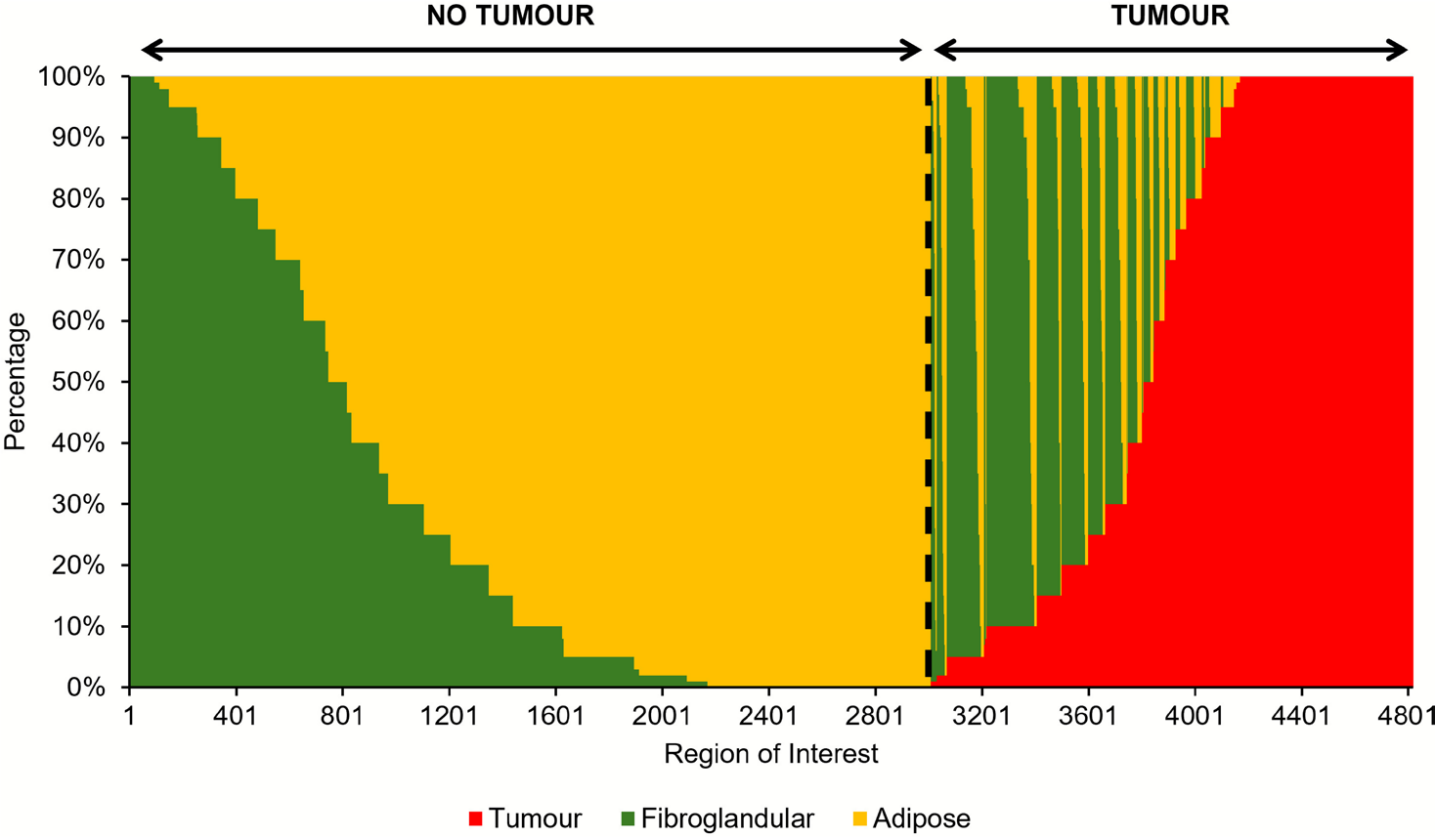
Hundred percent stacked column plots showing the distribution of tissue‐area percentages across all ROIs. Color corresponds to tissue type where red indicates tumor, green indicates fibroglandular, and yellow indicates adipose tissue. The black dashed line separates tumor and no tumor ROIs, further indicated by the respective arrows. Data are arranged in order of increasing tumor content.

In addition, only 33% of all measurements were homogeneous: 14% tumor, 2% fibroglandular, and 17% adipose. The remaining 67% were all heterogeneous measurements, where 24% of the dataset were considered heterogeneous tumor and 43% were heterogeneous no tumor. Figure 3 shows the distribution of tissue‐area percentages across all ROIs arranged in order of increasing tumor content. This dataset is largely heterogeneous with the majority of both tumor and no tumor ROIs containing adipose tissue. This is expected given breast tissue composition. High tumor ROIs are primarily homogeneous or contain some adipose, whereas low and medium tumor ROIs often include both fibroglandular and adipose tissue.

Prior work using homogeneous data concluded all 13 TRF‐DR features were beneficial for classifying the respective tissue types [20]. In this study, differences were observed between tumor and no tumor across all features, however, there was considerable overlap in the distributions (see Data S1). To reduce this, a base‐10 logarithm transform with PCA was applied to all optical features prior to machine learning. The corresponding PCs, associated variance, and feature loadings are summarized in Table 2. Larger loading magnitudes indicate features with strong correlations to the PC and the direction describes the relationship. Focusing on magnitude, PCs 1 and 2 explained ~61% of the total variance (39.8% and 20.9%, respectively) where the top 5 most strongly correlating features were the $\mu_{s}^{\prime }$ and DR values for (PC 1) and the $\mu_{a}$, normalized collagen intensity, and mean *τ*_FAD_ (PC 2). Stronger contributions from the TRF parameters were observed in PCs 3 and 4 with 24% of the total variance explained. The first four PCs accounted for 84.7% of the total variance and were deemed sufficient since including additional PCs did not greatly improve results (see Data S1). Compared to PC loadings from previous work [20], a similar trend was also observed.

**TABLE 2:** Summary of principal components, variance explained and associated feature loadings where the total variance is summed to 100%.

Components	Variance	$I_{\text{Collagen}}$	$I_{\mathrm{FAD}}$	$\tau_{\text{Collagen}}$	$\tau_{\text{NADH}}$	$\tau_{\mathrm{FAD}}$	DR 520 nm	DR 560 nm	$\mu_{a}$ 540 nm	$\mu_{a}$ 560 nm	$\mu_{a}$ 576 nm	$\mu_{s}^{\prime }$ 540 nm	$\mu_{s}^{\prime }$ 560 nm	$\mu_{s}^{\prime }$ 576 nm
PC1	0.398	0.195	−0.206	−0.120	0.123	0.045	0.382	0.384	−0.175	−0.162	−0.169	0.410	0.412	0.413
PC2	0.209	−0.238	0.175	0.012	0.140	0.250	−0.153	−0.031	−0.528	−0.482	−0.511	−0.119	−0.105	−0.094
PC3	0.164	−0.048	0.404	0.306	−0.605	−0.546	0.082	0.078	−0.126	−0.097	−0.127	0.086	0.087	0.088
PC4	0.076	−0.666	0.451	0.140	0.132	0.326	0.075	0.156	0.184	0.167	0.178	0.171	0.170	0.168
PC5	0.067	−0.344	0.002	−0.886	−0.229	−0.209	0.008	0.009	−0.008	−0.006	−0.008	−0.010	−0.009	−0.009
PC6	0.032	0.001	−0.045	−0.014	−0.021	0.001	−0.463	−0.575	−0.111	0.317	−0.227	0.307	0.310	0.312
PC7	0.023	−0.579	−0.673	0.287	0.002	−0.261	0.197	−0.128	−0.048	−0.018	−0.053	−0.018	−0.021	−0.024
PC8	0.019	−0.020	−0.037	0.008	−0.017	−0.026	−0.227	−0.233	0.210	−0.754	0.470	0.121	0.140	0.153
PC9	0.007	0.074	0.233	−0.063	0.108	0.030	0.709	−0.645	−0.018	−0.052	−0.007	−0.004	−0.021	−0.034
PC10	0.004	0.025	−0.223	0.024	−0.719	0.646	0.105	−0.055	0.005	−0.006	0.008	−0.005	−0.005	−0.005
PC11	0.000	0.002	0.001	0.000	0.001	−0.003	0.025	−0.007	0.012	0.023	−0.005	−0.740	0.060	0.669
PC12	0.000	0.000	0.000	0.000	0.000	0.000	0.000	0.000	0.763	−0.176	−0.622	0.006	−0.001	−0.006
PC13	0.000	0.000	0.000	0.000	0.000	0.000	0.000	0.000	0.000	0.000	0.000	0.352	−0.814	0.463

*Note:* This was performed on the dataset after base‐10 log transform and standardization. Shaded values highlight the top five most strongly correlating features where intensity corresponds to magnitude, for the respective PC. Only the top four PCs were used in the reported results.

Groups using similar DR and intrinsic fluorescence spectroscopy techniques reported DR features provided the best performance and the latter only offered slight improvements [30, 32, 40]. However, conclusions about fluorescence intensity and lifetime features are still unclear, for example, in improving overall classification accuracy [30, 32, 41, 42]. It has been suggested that these features may offer improved separation for identifying benign lesions (e.g., fibrocystic change) and fibrous, glandular tissue specifically [30, 31, 40]. The reported PC weightings suggest that DR features could be the most beneficial for heterogeneous breast tissue assessment. However, this may be due to inherent intra‐patient variations as studies reported $\mu_{a}$ and $\mu_{s}^{\prime }$ fluctuating as much as 20%–40% and 5%–20%, respectively, for the 660–980 nm range in healthy patients [43, 44]. Previous work also reported $\mu_{a}$ was not different between adipose and fibroglandular and that differences in $\mu_{s}^{\prime }$ between tumor and fibroglandular may be due to variance [19].

**FIGURE 4:**
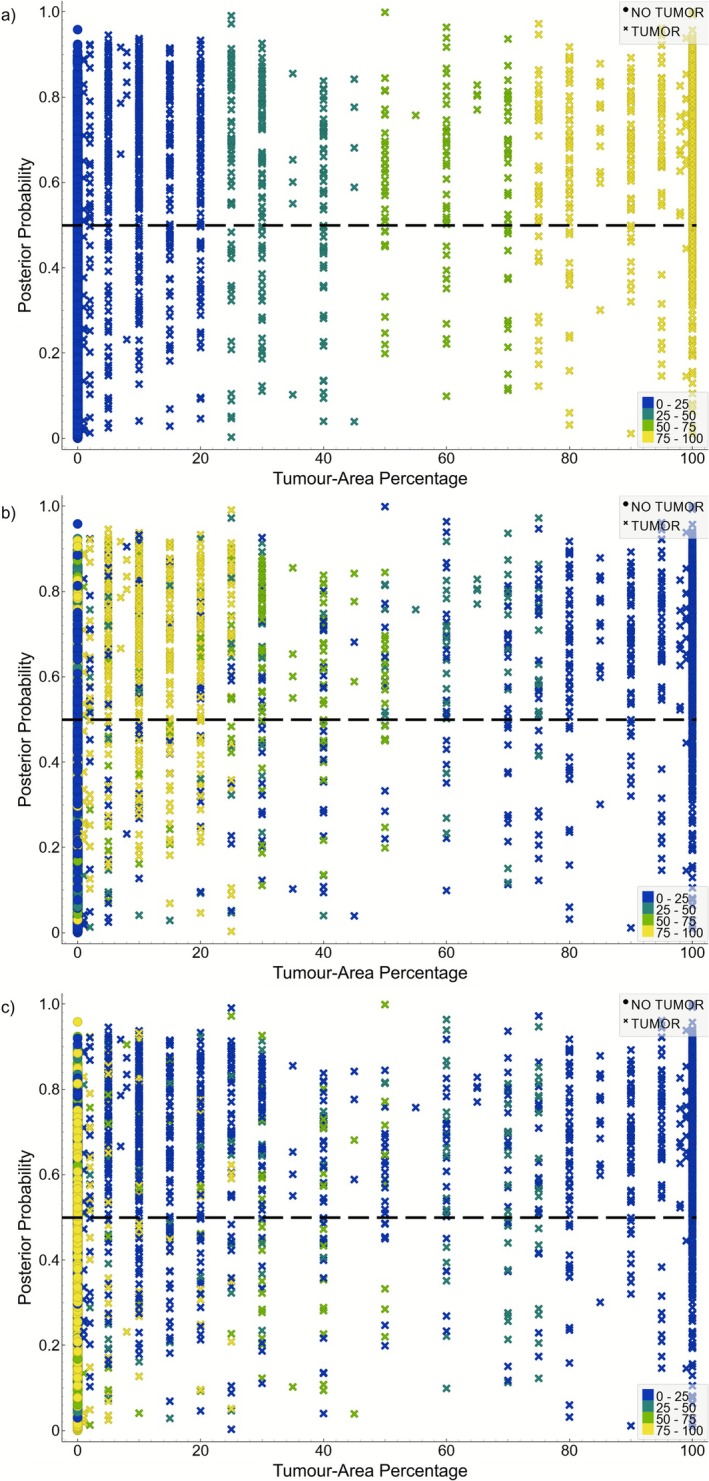
Posterior probabilities for each ROI arranged in order of increasing tumor‐area percentage. Color grading represents the distribution of pathologist‐assigned tissue percentages across the posterior probabilities for tumor (a), fibroglandular (b), and adipose (c). The color gradient is as follows: 0%–25% (dark blue), 25%–50% (dark green), 50%–75% (light green), and 75%–100% (yellow). The black dashed line represents the 50% tumor decision boundary used by the model. Datapoints above this threshold were predicted tumor and below no tumor. The corresponding shapes represent the pathologist assigned tissue class labels. Crosses above the decision boundary represent true positives and circles below this threshold are true negatives.

Using the top four PCs as feature inputs, a total of 2362 ROIs were predicted as tumor of which 59% were correctly classified. This contrasts with the 2456 no tumor predictions where 83% were accurate (Table 3). This resulted in a sensitivity and specificity of 77% and 68%, respectively (Table 4). The scatter plots in Figure 4 show how the tissue‐area percentages for tumor (Figure 4a), fibroglandular (Figure 4b), and adipose (Figure 4c) are distributed across the posterior probabilities. For the correct tumor predictions, 52% of these regions contained high amounts of tumor, 17% contained medium amounts, and 31% contained low amounts (Figure 4a). For the correct no tumor predictions, 11% contained high fibroglandular content (Figure 4b) and 73% contained high adipose content (Figure 4c). This indicates that many of the correct predictions are dominated by regions with high amounts of tumor or adipose tissue.

**TABLE 3:** Confusion matrix for classification of tumor and no tumor regions for the total dataset.

		Predicted		
		Tumor	No tumor	∑
Actual	Tumor	1399	413	1812
	No tumor	963	2043	3006
	∑	2362	2456	4818

*Note:* Shaded regions indicate correct class predictions.

**TABLE 4:** Summary of sensitivity and specificity across total, heterogeneous‐only, and tumor subsets.

Classification	Sensitivity	Specificity
Tumor vs. no tumor	0.772	0.680
Tumor vs. no tumor (heterogeneous‐only)	0.707	0.640
Low tumor vs. no tumor	0.737	0.670
Medium tumor vs. no tumor	0.717	0.642
High tumor vs. no tumor	0.806	0.702

*Note:* Results are from using four PC's as inputs with the model trained independently on each dataset.

In terms of incorrect predictions there were 963 false positive (FP) predictions. From Figure 4b,c, 33% and 42% of FPs contained high fibroglandular and adipose content, respectively. For the low and medium tissue percentages of both fibroglandular and adipose, these were found to contribute approximately equal amounts to FP predictions: 28% and 24% (low) and 28% and 28% (medium). This demonstrates that FP predictions are also heavily influenced by high adipose tissue presence with a greater contribution from high fibroglandular compared to the correct no tumor predictions (i.e., 33% vs. 11%). For the 413 false negative (FN) predictions, 28% contained only tumor, 24% contained medium amounts, and 37% were low tumor percentages (Figure 4a). Of the low and medium tumor amounts, 53% and 37% contained regions with all tissue types, respectively. Out of all FN predictions, 23% contained high amounts of fibroglandular and 9% high adipose tissue. Compared to the FP predictions, the FN predictions largely came from ROIs with high fibroglandular tissue. Furthermore, even though high tumor amounts were the most prevalent in the correct true predictions, the model still misclassified a substantial portion of these ROIs. Overall, of the homogeneous regions, 83% were correctly predicted, where 82% tumor, 37% fibroglandular, and 88% adipose were accurately classified. Of the heterogeneous measurements, 66% were accurate with 74% and 61% of the tumor and no tumor regions being correctly assigned. Furthermore, of the heterogeneous tumor regions, 75% low, 70% medium, and 81% high were correctly classified. When only comparing low, medium, and high tumor‐area regions against no tumor separately (Table 4), sensitivities and specificities ranged from 72% to 81% and 64% to 70%, respectively (Table 4).

Prior work has demonstrated the system's ability to separate tumor from fibroglandular and adipose tissue in homogeneous regions [20]. It is encouraging that most homogeneous regions were still correctly identified despite having a heterogeneous dataset. In instances of regions comprised entirely of tumor, 82% were correctly identified, comparable with adipose. However, of regions entirely comprised of fibroglandular tissue, only 37% were correctly classified as no tumor. This is expected when 2% of the data contained only fibroglandular tissue. If only assessing heterogeneous regions, the sensitivity and specificity decrease to 71% and 64%, respectively (Table 4). This highlights the influence of homogeneous tumor and adipose regions on model training and is further supported when only assessing high tumor separation compared with low and medium tumor separation (Table 4).

This work also demonstrates the challenges of accurately detecting tumor in the presence of fibroglandular and adipose tissue of varying levels. Consequently, this resulted in FP (32%) and FN (23%) rates (total dataset) that are either comparable to (e.g., specimen radiography—FP rate: 16%; FN rate: 47%) or much greater (e.g., frozen section analysis—FP rate: 4%; FN rate: 14%) than other modalities [45]. As observed, many FPs and FNs are due to high fibroglandular presence. This was expected when considering the difficulty in visually separating normal fibrous tissue from tumor [25, 31], with this also being reflected in optical parameter values [19, 28, 35]. However, it was unexpected that 82% of the correct heterogeneous low tumor ROIs also contained high fibroglandular presence (Figure 4b). This shows that the effects of fibroglandular tissue can extend beyond misclassifications, making it difficult to define a ‘minimum tumor detection limit’ for this system. Attempts were made by parsing the dataset further into low, medium, and high tumor amounts and having the model trained separately. Results from Table 4 demonstrate the model achieved the highest sensitivity and specificity for high tumor (sensitivity: 81%; specificity: 70%) separation, followed by low tumor (sensitivity: 74%; specificity: 67%) and medium tumor (sensitivity: 72%; specificity: 64%) separation. One reason for low tumor performance being greater than medium tumor is having nearly double the ROIs (Table 1). However, as already highlighted, many of the correct low tumor predictions contained high fibroglandular presence; therefore, it is unclear whether the model is accurately distinguishing tumor from fibroglandular tissue in low quantities. A study using DR demonstrated that certain features were statistically different between IDC and glandular tissue; however, only when tumor presence was > 50% [28]. Compared to this study, the current system can classify tumor in amounts ≥ 75% but no concrete conclusions can be drawn for low to medium tumor predictions.

Interestingly, the specificity for these subsets also followed similar trends. For the high tumor subset, even with this class being mostly homogeneous, this was insufficient for offsetting the effects of heterogeneous normal tissue. This can be observed by the significant portion of FPs from regions with high adipose (Figure 4c). Few studies have reported that adipose mixed with tumor [29, 46] or in lesser amounts [24] can lead to misclassifications and relatively poorer performance. These findings suggest that under mixed tissue environments, adipose is difficult to differentiate.

Finally, reasons for why 28% of the FNs contained homogeneous tumor are unknown but may be attributed to the inherent patient characteristics of this dataset (see Data S1). Although incomplete, patients contained either invasive ductal or lobular carcinomas, with some also found to contain IDC, DCIS, along with fibrocystic change and fibroadenoma within a single specimen. Studies which performed subtype classifications, although obtained good performance, demonstrated overlap in optical features between the subclasses [28, 30, 35, 46]. This can complicate model training due to the gradual change in tissue composition from normal, benign, and malignant lesions, further made difficult by the amount of each type present within a region [28, 35].

Compared to findings from Majumder et al., which used comparable wavelengths and sparse multinomial logistic regression, rates for misclassifying tumor and FPs were either comparable or ~10% less to what was achieved in this study [46]. Furthermore, there was greater spread in the posterior probabilities across normal tissue predictions compared with other subtypes, with many misclassified normal tissue regions having predominantly fibrous or glandular tissue compared with adipose. This study assigned the dominant tissue type for histopathology; thus, it is encouraging that our results follow similar observations using heterogeneous data. This effect of fibrous (connective) tissue on model predictions has also been observed when using near‐infrared DR spectral features and a weighted support vector machine (SVM) on mixed tissue [34]. To our knowledge, this is the only other study similarly analyzing the effect of heterogeneous measurements on optical features and model predictions. Multiple variants of the weighted SVM were trained on increasing thresholds of connective and adipose tissue in 10% increments for determining whether a site should be labeled tumor or healthy [34]. Interestingly, using an independent set, it found models trained on tumor regions with ≤ 10% adipose tissue and healthy regions with ≤ 20% connective tissue performed comparably to the model trained on homogeneous regions. However, models trained with greater adipose and/or connective tissue thresholds performed worse, and results on the independent test set were not provided. The findings suggest that heterogeneous tissue training may not be necessary; however, the independent test set contained largely fat with insufficient tumor and connective tissue amounts [34]. Therefore, it is difficult to determine how including greater heterogeneity in the test set would have influenced model predictions. Ultimately, comparing results for different systems is impossible due to differing source geometries, excitation and emission wavelengths, sample compositions, feature extraction methods, and choice of machine learning algorithm(s).

Overall, these results suggest the TRF‐DR spectroscopy system combined with weighted logistic regression can delineate tumor from normal tissue on heterogeneous measurements. This is supported by a sensitivity and specificity comparable to other commercially available techniques (e.g., MarginProbe—sensitivity: 70.1%; specificity: 47.5%) [47]. However, the high false positive and FN rates due to the difficulty in identifying tumor in low or medium amounts and adipose in mixed amounts may affect treatment outcomes. For example, patients with smaller breasts may suffer worse cosmesis due to removing more tissue than necessary [47]. Furthermore, the difficulty in separating fibroglandular from tumor tissue may warrant caution in using this system on patients with denser breast. There is also the increased risk of local recurrence and additional surgery due to the high FN rate. These findings establish a baseline system performance in heterogeneous measurements for future work to improve upon. When considering advantages such as fast point‐measurement acquisitions (~5 s), intuitive operation, portability, and affordability, these results support the value of optical techniques for breast margin assessment.

Given the nature of this dataset, there is the confounding effect of inter‐patient heterogeneity (e.g., varying number of ROIs per validation fold). Furthermore, having incomplete patient characteristics make it difficult to correlate with model predictions to draw meaningful relationships. There are also limited sample measurements and the use of a single pathologist for tissue annotations. Compared with our prior study, the sample size is larger but still too small for an independent test set. Consequently, the models' decision boundary could not be further optimized. There are also reproducibility concerns around quantifying tissue‐area percentages by relying on a single pathologist, as this is likely to vary between observers, specifically in low tumor regions. The sample collection protocol, although provides more datapoints, are from specific sections of the gross specimen. These may not reflect the full extent of tissue heterogeneity across patients.

Future work will aim to improve upon these limitations through collecting new frozen and fresh breast sample measurements in a gross specimen suite. This will create an independent test set to evaluate generalizability of the current model and parameters along with any future changes to methodology. Such changes will include (1) multiple pathologists to assess inter‐observer variability; (2) more standardized image registration and tissue‐area percentage algorithms using third‐party software; and (3) further exploring tissue heterogeneity and developing methods for improving system robustness to such effects. More specifically, this will involve using alternative features (e.g., autofluorescence intensity spectra) and Monte Carlo simulations to better capture heterogeneous tissue dynamics, methods for both intra‐ and inter‐patient normalization, alternative machine learning models such as multinomial logistic regression [29, 30, 46] for improving low and medium tumor predictions and to systematically identify a “minimum tumor detection limit.” In addition, more complex algorithms such as random forest and its variants (e.g., extreme gradient boost) or artificial neural networks will also be explored as these models have been successfully applied to this problem by other groups [24, 26, 27], with the trade‐off of model interpretability. In creating an independent test set, these models may be more robust to dataset differences and be better suited for such heterogeneous, overlapping data when provided sufficient samples.

## 4 Conclusion

Breast cancer margin width definitions are still not standardized, and a variety of approaches have been developed or are underway to offer more quantitative intraoperative margin assessment tools. This study demonstrates the potential for the TRF‐DR system to function as a complementary margin assessment device, namely for direct sensing of the tumor margin on the excised lump. We performed a deliberate investigation of heterogeneous composition and its effects on the distribution of optical features and machine learning using frozen breast tissue samples. From PCA, it was found that DR features explained most of the variance compared to TRF features. This corresponds with previous findings and follows similar trends to other groups, suggesting DR may be more suitable for handling heterogeneous regions. Furthermore, evaluating the posterior probabilities from the logistic regressor allowed for semi‐quantitative analysis of the effects of fibroglandular and adipose tissue on model predictions. It was found that many of the FPs and FNs were due to high fibroglandular and adipose presence in regions containing tumor. Although the difficulty in separating tumor from fibroglandular tissue are known, these results provide further context into how this can affect machine learning predictions. It also highlights the difficulty in identifying adipose when not present in high amounts. Similar trends were also observed when stratifying model training into low, medium, and high tumor. These results stress that efforts must be made directly toward improving tumor‐fibroglandular separation when developing similar techniques. Likewise, these findings present numerous avenues for further development of our system in the form of (1) improved image registration and standardized tissue‐area percentage calculations; (2) exploring different features and extraction methods (e.g., autofluorescence analysis, Monte Carlo simulations) that may improve model performance, specifically in separating fibroglandular tissue; and (3) collecting independent test data using frozen and fresh tissue in a gross specimen suite to assess clinical feasibility.

## Author Contributions

J.L. and M.J.F., conceived the study. J.L., Q.F., T.J.F., and M.J.F. developed study methodology. E.D. performed measurement collection, dataset preprocessing, developed code for optical parameter extraction, provided images for Figure 2. P.L. provided tissue samples and patient data. G.G. provided tissue classification and tissue‐area percentages. J.L. performed data preprocessing, analysis, and prepared all figures. All authors contributed to writing and editing of the manuscript and provided final approval prior submission.

## Funding

This work was supported by the Natural Sciences and Engineering Research Council of Canada and Canada Foundation for Innovation.

## Ethics Statement

Study approval for sample collection and analysis was granted by the Hamilton Integrated Research Ethics Board (Project No. 10‐3393) at St. Joseph's Hospital, Hamilton, Ontario. All procedures necessary for the secure handling of patient data were in accordance with Hamilton Integrated Research Ethics Boards policies.

## Consent

Informed consent was obtained from all individuals included in this study. Patient consent was waived by the ethics board as information was anonymized for the study.

## Conflicts of Interest

The authors declare no conflicts of interest.

## Supporting information


**Data S1:** jbio70230‐sup‐0001‐Supinfo.docx.

## Data Availability

The data that support the findings of this study are available from the corresponding author upon reasonable request.
