# Supplementary material for: Time‐Resolved Fluorescence and Diffuse Reflectance (TRF‐DR) Spectroscopy for Heterogeneous Breast Tissue Classification and Tumor Margin Assessment
Source: J Biophotonics. 2026 Feb 1;19(2):e70230. doi: 10.1002/jbio.70230 (PMC12862050; doi:10.1002/jbio.70230)
Supplement: Supplementary file 1 — Data S1: jbio70230‐sup‐0001‐Supinfo.docx. [file JBIO-19-e70230-s001.docx]

**Supplemental Material**

*Supplemental 1:* Characteristics of patient cohort. Important to note that information for 15 patients is missing.

| *Patients* | | 57 |
| --- | --- | --- |
| *Mean Age (Range)* | | 67 (42-87) |
| *Menopausal Status* | |  |
|  | Premenopausal | 7 |
|  | Perimenopausal | 0 |
|  | Postmenopausal | 50 |
| *Procedure Type* | |  |
|  | Lumpectomy | 39 |
|  | Mastectomy | 18 |
| *Mean Specimen volume (std) [cm^3^]* | | 754.4 (1208.5) |
| *Mean Tumour Size (std) [cm]* | | 3.7 (2.0) |
| *Histological Type* | |  |
|  | Ductal | 44 |
|  | Lobular | 10 |
|  | Metaplastic carcinoma | 2 |
|  | Mixed | 1 |
| *Invasive Tumour Grade* | | G1:4; G2:30; G3:23 |
| *Ductal carcinoma in situ* | | 36 |
| *Extensive Intraductal Component* | | 6 |
| *Lymphovascular Invasion* | | 18 |
| *Multifocal Disease* | | 4 |
| *Lobular carcinoma in situ* | | 8 |
| *Local Tumour Stage* | | T1:8; T2:40; T3:9; T4:0 |
| *Nodal Status* | | N0:36; N1:14; N2:3; N3:3; NX:1 |
| *Receptor Status* | |  |
|  | ER+PR+HER2+ | 5 |
|  | ER+PR+HER2- | 36 |
|  | ER+PR-HER2- | 3 |
|  | ER+PR-HER2+ | 4 |
|  | ER-PR-HER2+ | 1 |
|  | ER-PR-HER2- | 6 |
|  | ER+PR-NOS | 2 |

NOS Not otherwise specified


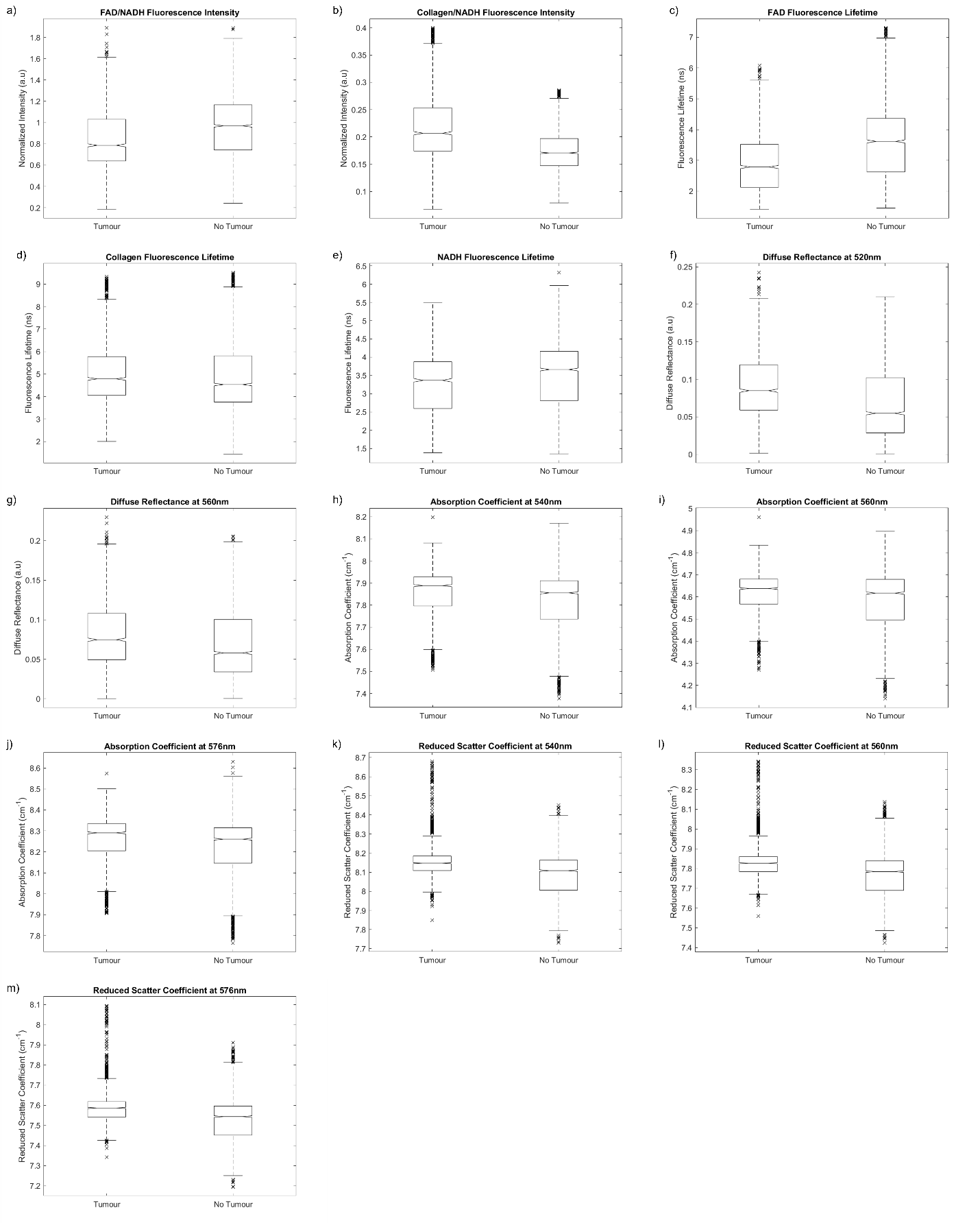


*Supplemental 2:* Box plots of normalized fluorescence intensities (a, b), mean fluorescent τ (c, d, e), DR at wavelengths 520 nm (f) and 560 nm (g), $\mu_{a}$ at wavelength 540 nm (h), 560 nm (i), 576 nm (j), and $\mu_{s}^{'}$ at wavelength 540 nm (k), 560 nm (l), 576 nm (m). All distributions were statistically significantly different (*p <* 0.05) according to the Mann-Whitney U test.

*Supplemental 3:* Summary of mean and standard error for time-resolved fluorescence and diffuse reflectance features for tumour and no tumour groups.

| *Features* | *Tumour (n = 1812)* | *No Tumour (n = 3006)* |
| --- | --- | --- |
| Time-resolved Fluorescence |  |  |
| Mean $I_{Collagen}$ | 0.219 ± 0.001 | 0.1742 ± 0.0007 |
| Mean $I_{FAD}$ | 0.844 ± 0.006 | 0.957 ± 0.005 |
| $\tau_{Collagen}$ (ns) | 5.05 ± 0.03 | 4.88 ± 0.03 |
| $\tau_{NADH}$ (ns) | 3.20 ± 0.02 | 3.50 ± 0.02 |
| $\tau_{FAD}$ (ns) | 2.89 ± 0.02 | 3.62 ± 0.02 |
| Diffuse Reflectance |  |  |
| DR 520 nm | 0.091 ± 0.001 | 0.0690 ± 0.0009 |
| DR 560 nm | 0.081 ± 0.001 | 0.0699 ± 0.0008 |
| $\mu_{a}$ 540 nm (cm^-1^) | 7.854 ± 0.003 | 7.811 ± 0.003 |
| $\mu_{a}$ 560 nm (cm^-1^) | 4.615 ± 0.002 | 4.587 ± 0.002 |
| $\mu_{a}$ 576 nm (cm^-1^) | 8.260 ± 0.003 | 8.216 ± 0.003 |
| $\mu_{s}^{'}$ 540 nm (cm^-1^) | 8.164 ± 0.003 | 8.109 ± 0.002 |
| $\mu_{s}^{'}$ 560 nm (cm^-1^) | 7.841 ± 0.003 | 7.790 ± 0.002 |
| $\mu_{s}^{'}$ 576 nm (cm^-1^) | 7.599 ± 0.003 | 7.551 ± 0.002 |
| $I_{x}$ fluorescence intensity  $\tau_{x}$ fluorescence liftetime  DR diffuse reflectance  $\mu_{a}$ absorption coefficient  $\mu_{s}^{'}$ reduced scattering coefficient | | |

*Supplemental 4:* Summary of sensitivity and specificity with increasing number of PCs serving as inputs for the total dataset. For example, the results associated with PC1 represent model performance when only 1 PC was selected.

| *Components* | *Variance* | *Total Variance* | *Sensitivity* | *Specificity* |
| --- | --- | --- | --- | --- |
| PC1 | 0.398 | 0.398 | 0.664 | 0.552 |
| PC2 | 0.209 | 0.607 | 0.653 | 0.587 |
| PC3 | 0.164 | 0.771 | 0.778 | 0.655 |
| PC4 | 0.076 | 0.847 | 0.772 | 0.680 |
| PC5 | 0.067 | 0.914 | 0.769 | 0.679 |
| PC6 | 0.032 | 0.946 | 0.766 | 0.684 |
| PC7 | 0.023 | 0.969 | 0.765 | 0.691 |
| PC8 | 0.019 | 0.988 | 0.762 | 0.691 |
| PC9 | 0.007 | 0.995 | 0.760 | 0.691 |
| PC10 | 0.004 | 0.999 | 0.760 | 0.691 |
| PC11 | 0.000 | 1 | 0.758 | 0.693 |
| PC12 | 0.000 | 1 | 0.758 | 0.693 |
